# Genome sequences of three *Aegilops* species of the section Sitopsis reveal phylogenetic relationships and provide resources for wheat improvement

**DOI:** 10.1111/tpj.15664

**Published:** 2022-02-12

**Authors:** Raz Avni, Thomas Lux, Anna Minz‐Dub, Eitan Millet, Hanan Sela, Assaf Distelfeld, Jasline Deek, Guotai Yu, Burkhard Steuernagel, Curtis Pozniak, Jennifer Ens, Heidrun Gundlach, Klaus F. X. Mayer, Axel Himmelbach, Nils Stein, Martin Mascher, Manuel Spannagl, Brande B. H. Wulff, Amir Sharon

**Affiliations:** ^1^ Wise Faculty of Life Sciences, Institute for Cereal Crops Improvement and School of Plant Sciences and Food Security Tel Aviv University Tel Aviv 6997801 Israel; ^2^ Plant Genome and Systems Biology (PGSB) Helmholtz‐Center Munich Ingolstädter Landstraße 1 Neuherberg D‐85764 Germany; ^3^ Wise Faculty of Life Sciences, Institute for Cereal Crops Improvement Tel Aviv University Tel Aviv 6997801 Israel; ^4^ John Innes Centre Norwich Research Park Norwich NR4 7UH UK; ^5^ Department of Plant Sciences and Crop Development Centre, College of Agriculture and Bioresources University of Saskatchewan Campus Drive 51 Saskatoon S7N 5A8 Canada; ^6^ Faculty of Life Sciences Technical University Munich Weihenstephan Munich D‐80333 Germany; ^7^ Center of Integrated Breeding Research (CiBreed), Department of Crop Sciences Georg‐August‐University Von Siebold Str. 8 Göttingen 37075 Germany; ^8^ Leibniz‐Institute of Plant Genetics and Crop Plant Research (IPK) Gatersleben Corrensstrasse 3 Seeland 06466 Germany; ^9^ German Centre for Integrative Biodiversity Research (iDiv) Halle‐Jena‐Leipzig Puschstrasse 4 Leipzig D‐04103 Germany; ^10^ Present address: Leibniz Institute of Plant Genetics and Crop Plant Research (IPK) Gatersleben Corrensstrasse 3 Seeland 06466 Germany; ^11^ Present address: Department of Evolutionary and Environmental Biology, Faculty of Natural Sciences, Institute of Evolution University of Haifa 199 Aba Khoushy Ave., Mount Carmel Haifa 3498838 Israel; ^12^ Present address: Center for Desert Agriculture, Biological and Environmental Science and Engineering Division (BESE) King Abdullah University of Science and Technology (KAUST) Thuwal 23955‐6900 Saudi Arabia

**Keywords:** *Aegilops*, Sitopsis, genome sequence, annotation, nucleotide‐binding leucine‐rich repeat (NLR), haplotype

## Abstract

*Aegilops* is a close relative of wheat (*Triticum* spp.), and *Aegilops* species in the section Sitopsis represent a rich reservoir of genetic diversity for the improvement of wheat. To understand their diversity and advance their utilization, we produced whole‐genome assemblies of *Aegilops longissima* and *Aegilops speltoides*. Whole‐genome comparative analysis, along with the recently sequenced *Aegilops sharonensis* genome, showed that the *Ae. longissima* and *Ae. sharonensis* genomes are highly similar and are most closely related to the wheat D subgenome. By contrast, the *Ae. speltoides* genome is more closely related to the B subgenome. Haplotype block analysis supported the idea that *Ae. speltoides* genome is closest to the wheat B subgenome, and highlighted variable and similar genomic regions between the three *Aegilops* species and wheat. Genome‐wide analysis of *nucleotide‐binding leucine‐rich repeat* (*NLR*) genes revealed species‐specific and lineage‐specific *NLR* genes and variants, demonstrating the potential of *Aegilops* genomes for wheat improvement.

## Significance Statement

Whole‐genome comparative analysis using *de novo* assemblies of *Aegilops longissima* and *Aegilops speltoides*, along with the recently sequenced *Aegilops sharonensis* genome, strengthen evolutionary evidence that *Ae. longissima* and *Ae. sharonensis* genomes are highly similar and are most closely related to the wheat D subgenome. By contrast, the *Ae. speltoides* genome is more closely related to the B subgenome. A detailed NLR annotation and haplotype block analysis aims to help further research using these species to improve wheat.

## INTRODUCTION

Wheat domestication started some 10 000 years ago in the Southern Levant with the cultivation of wild emmer wheat (WEW): *Triticum turgidum* subsp. *dicoccoides* (Koern. ex Asch. & Graebn.) Thell. (genome AABB) (Zohary et al., 2012). Expansion of cultivated emmer to other geographical regions, including Transcaucasia, allowed its hybridization with *Aegilops tauschii* (genome DD) and resulted in the emergence of hexaploid bread wheat: *Triticum aestivum* L. ssp. *aestivum* (genome AABBDD) (Giles and Brown, 2006). Over the next 8500 years, bread wheat spread worldwide to occupy nearly 95% of the 215 million hectares devoted to wheat cultivation today (Mastrangelo and Cattivelli, 2021). The success of bread wheat has been attributed at least in part to the plasticity of the hexaploid genome, which allowed wider adaptation compared with tetraploid wheat (Dubcovsky and Dvorak 2007). However, further improvement of wheat is limited by its narrow genetic diversity, a result of domestication and the limited number of hybridization events from which hexaploid wheat evolved (Bernhardt et al., 2020; Gaurav et al., 2021; Reif et al., 2005; Zhou et al., 2020).

To overcome the limited primary gene pool of wheat, breeders have used wide crosses with wild wheat relatives, which contain a rich reservoir of genetic diversity. Although many useful traits have been introgressed into wheat over the years (Pont et al., 2019), genetic constraints limit wide crosses to species that are phylogenetically close to wheat. Furthermore, some species in the secondary and tertiary gene pools of wheat (including *Aegilops longissima* and *Aegilops sharonensis*) cause chromosome breakage and preferential transmission of undesired gametes in wheat hybrids through the presence of so‐called gametocidal genes, which restrict interspecific hybridization (Finch et al. 1984; Tsujimoto, 1994). Therefore, introgression requires complex cytogenetic manipulations (Khazan et al., 2020; Kilian et al., 2011) and extensive backcrossing to recover the desired agronomic traits of the recipient wheat cultivar. These limitations can be overcome by using gene editing and genetic engineering technologies, which are not limited by plant species and can augment genetic crosses and substantially expedite the transfer of new traits into elite wheat cultivars (Arora et al., 2019; Luo et al., 2021; Uauy et al., 2017). Such technologies require high‐quality genomic sequences and gene annotation, which are essential for the evaluation of diversity in the respective wild species and for the efficient molecular isolation of candidate genetic loci.

*Aegilops* is the closest genus to *Triticum*, and species within this genus are considered ancestors of the wheat B and D subgenomes (Wang et al., 2013). The progenitor of the D subgenome of bread wheat is *Ae. tauschii* (Luo et al., 2017), which has a homologous D genome and can be readily crossed with tetraploid and hexaploid wheat (Kishii, 2019). *Aegilops* species in the section Sitopsis contain homoeologous S or S* genomes that are also closely related to wheat; however, their relationships with specific wheat subgenomes are less clear. Initially, each of the five Sitopsis members were considered potential progenitors of the wheat B subgenome (Kerby and Kuspira, 1987), but later studies showed that *Aegilops speltoides* (genome S) occupies a basal evolutionary position and is closest to the wheat B subgenome, whereas the other four species (genome S*) seem more closely related to the wheat D subgenome (Marcussen et al., 2014; Petersen et al., 2006;The International Wheat Genome Sequencing Consortium [IWGSC], 2014; Yamane and Kawahara, 2005). More recent analyses suggest separating *Ae. speltoides* from the other Sitopsis species, placing it phylogenetically together with *Amblyopyrum muticum* (diploid T genome) (Bernhardt et al., 2020; Edet et al., 2018; Glémin et al., 2019; Huynh et al., 2019).

The *Aegilops* species in the Sitopsis section contain many useful traits, in particular for disease resistance and abiotic stress tolerance (Anikster et al., 2005; Huang et al., 2018; Olivera et al., 2007; Scott et al., 2014). However, because the crossing of species with S and S* genomes to wheat is not straightforward, to date, only a handful of genes have been transferred from Sitopsis species to wheat. Better genomic tools and, in particular, high‐quality S genome sequences will provide a deeper understanding of the genomic relationships of these species to other wheat species and enhance efforts to identify and isolate useful genes.

In this study, we generated reference‐quality genome assemblies of *Ae. longissima* and *Ae. speltoides* and performed comparative analyses of these genomes together with the recently assembled *Ae. sharonensis* genome (Yu et al., 2021) to determine the evolutionary relationships between these Sitopsis species and wheat. Whole‐genome analysis of genes encoding nucleotide‐binding leucine‐rich repeat (NLR) factors, which play key roles in disease resistance, revealed species‐specific and lineage‐specific *NLR* genes and gene variants, highlighting the potential of these wild relatives as reservoirs for novel resistance genes for wheat improvement.

## RESULTS

### Assembly details and genome alignments

**Figure 1:**
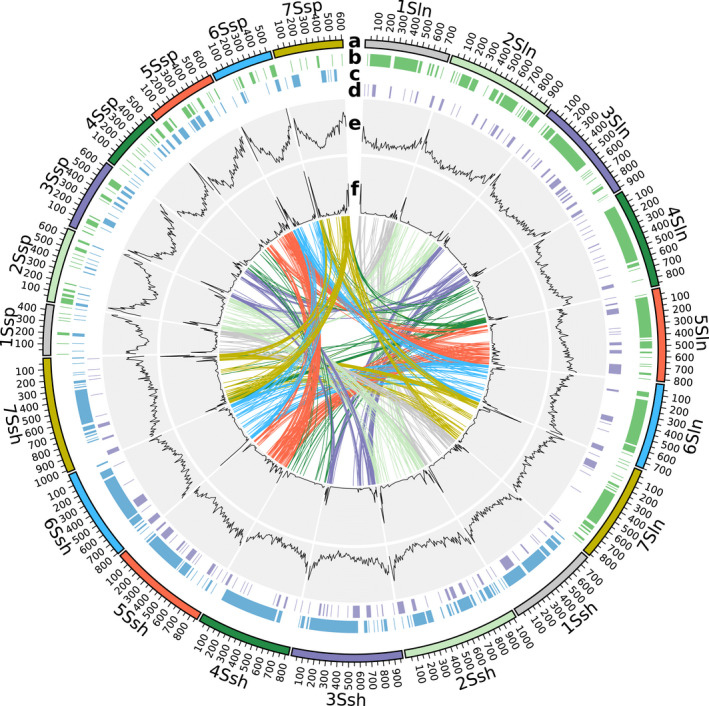
Chromosome‐scale assembly and annotation of the *Aegilops longissima*, *Aegilops sharonensis* and *Aegilops speltoides* genomes: a, chromosomes*; b, haploblocks between *Ae. sharonensis*/*Ae. longissima* and *Ae. sharonensis*/*Ae. speltoides*; c, haploblocks between *Ae. longissima*/*Ae. sharonensis* and *Ae. longissima*/*Ae. speltoides*; d, haploblocks between *Ae. speltoides*/*Ae. longissima* and *Ae. speltoides*/*Ae. sharonensis*; e, distribution of all genes; f, distribution of *NLR* genes. Connecting lines show links between orthologous *NLR* genes. *The title is composed of the chromosome number, genome (S) and the species: *Ae. longissima* (ln), *Ae. sharonensis* (sh) and *Ae. speltoides* (sp).

We used a combination of Illumina (https://www.illumina.com) 250‐bp paired‐end reads, 150‐bp mate‐pair reads, and 10x Genomics (https://www.10xgenomics.com) and Hi‐C libraries for sequencing of high‐molecular‐weight DNA and chromosome‐level assembly of the *Ae. longissima* and *Ae. speltoides* genomes. Pseudomolecule assembly using the tritex pipeline (Monat et al., 2019) yielded a scaffold *N50* (the sequence length of the shortest contig at 50% of the total genome length) of 3 754 329 bp for *Ae. longissima* and 3 111 390 bp for *Ae. speltoides* (Tables S1 and S2), and all three genomes assembled into seven chromosomes, as expected in diploid wheat. The genome of *Ae. longissima* has an assembly size of 6.70 Gb, highly similar to that of *Ae. sharonensis* (6.71 Gb), and substantially larger than the 5.13‐Gb assembly of *Ae. speltoides* (Figure 1a). These values are in agreement with nuclear DNA quantification that showed nuclear DNA contents (C‐values) of approximately 7.5, 7.5 and 5.8 pg for *Ae. longissima*, *Ae. sharonensis* and *Ae. speltoides*, respectively (Eilam et al., 2008). GC content and transposable element composition were analysed in comparison with bread wheat subgenomes and showed very similar results (Tables S3 and S4).

**Figure 2:**
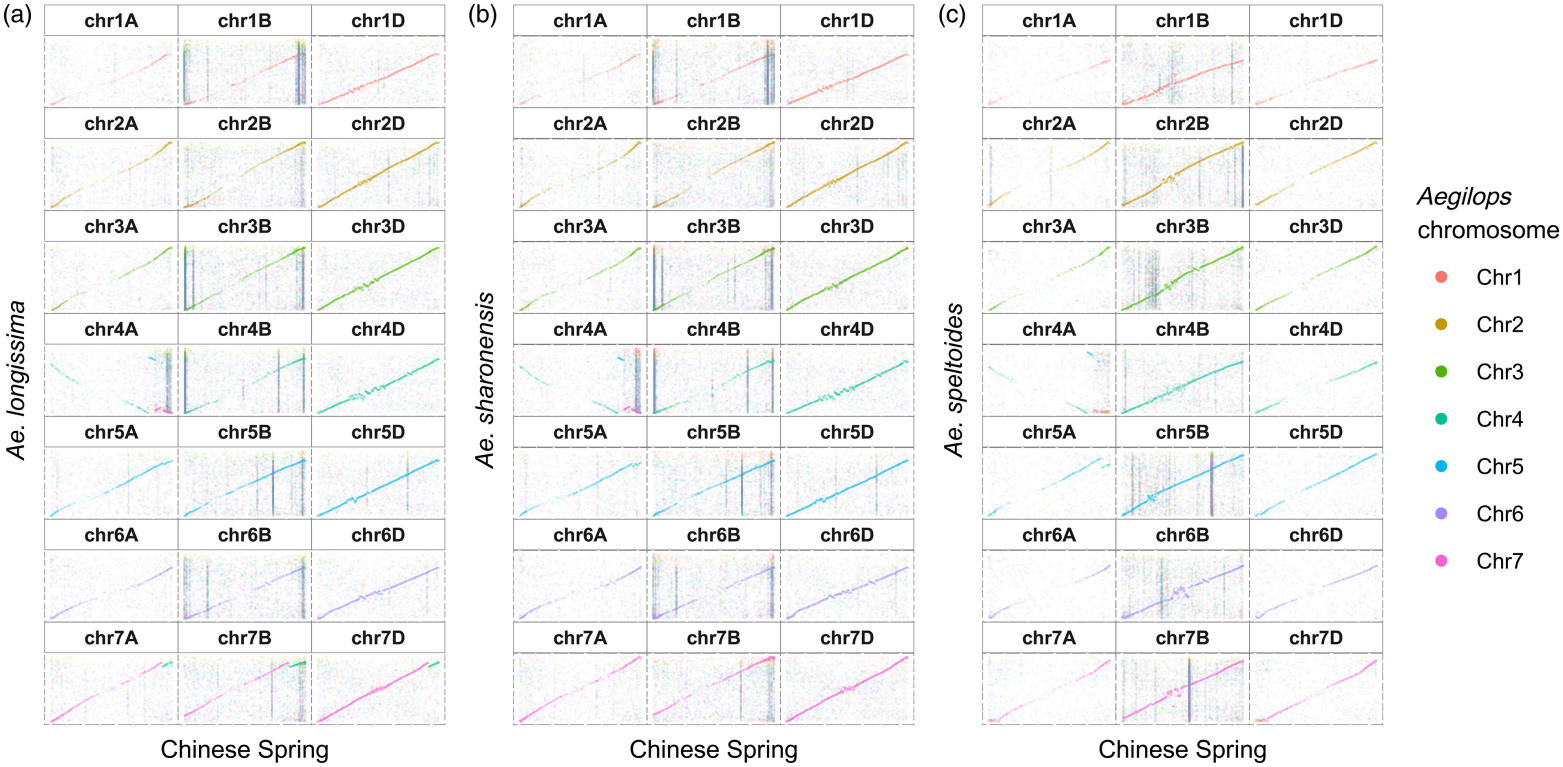
Whole‐genome alignments. Alignments between *Triticum aestivum* cv. Chinese Spring (*x*‐axis) and the three *Aegilops* species (*y*‐axis): (a) *Aegilops longissima*; (b) *Aegilops sharonensis*; (c) *Aegilops speltoides*. The best alignments are between *Ae. longissima* and *Ae. sharonensis* and the *T. aestivum* cv. Chinese Spring D subgenome, and between *Ae. speltoides* and the *T. aestivum* cv. Chinese Spring B subgenome. The vertical lines represent repetitive sequences (for example in panel c, chr7B). A previously reported translocation in *Ae. longissima* between chromosomes 7S and 4S can be seen in (a) as a teal‐colored segment (approx. 54 Mb) at the end of the long arm of group‐7 chromosomes of ‘Chinese Spring’.

The structural integrity of the pseudomolecule assemblies of *Ae. longissima* and *Ae. speltoides* was validated by inspection of Hi‐C contact matrices (Figure S1). A busco (Simão et al., 2015) analysis was conducted to assess the genome assemblies and annotation qualities. This analysis showed a high level of genome completeness, with 97.8% for *Ae. sharonensis*, 97.5% for *Ae. longissima* and 96.4% for *Ae. speltoides* (Figure S2). The chromosome sizes in *Ae. longissima* and *Ae. sharonensis* were similar for all chromosomes, except for chromosome 7, which is much smaller in *Ae. longissima*, in part as a result of translocation to chromosome 4. The size of the translocation as measured from the whole‐genome alignment is approximately 54 Mb (Zhang et al., 2001) (Figure 2a).

### Gene space annotation and ortholog analysis

We generated gene annotations for *Ae. longissima* and *Ae. speltoides* based on sequence analysis and homology to other plant species and used the same approach with the addition of RNA‐seq data to generate gene annotation for the recently sequenced *Ae. sharonensis* assembly (Yu et al., 2021). Details of all annotations are presented in Table S1. There are 36 928 high‐confidence genes in *Ae. speltoides*, approximately 20% higher than the estimated 31 183 genes in *Ae. longissima* and 31 198 genes in *Ae. sharonensis*. The gene density in all three genomes is higher near the telomeres (Figure 1e), similar to what has been observed in other Triticeae species (Avni et al., 2017; IWGSC et al., 2018; Luo et al., 2017). As *Ae. speltoides* reproduces by cross‐pollination, the higher number of predicted genes compared with the other two species may reflect a higher degree of heterozygosity. This notion is supported by the relatively large number of *Ae. speltoides* genes that are found on scaffolds that were not assigned to a chromosome (Table S1).

**Figure 3:**
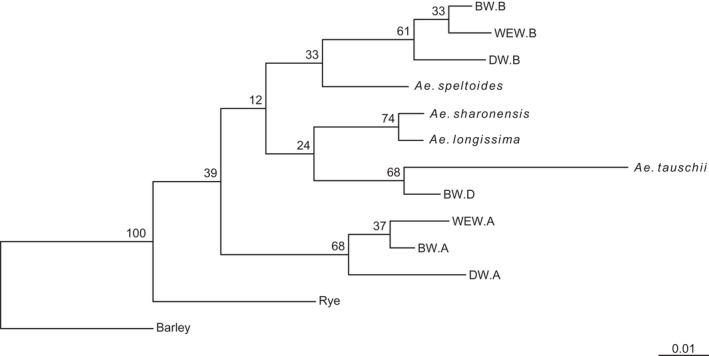
Phylogenetic tree based on orthofinder analysis of all high‐confidence genes. Branch values correspond to orthofinder support values. BW, bread wheat cv. Chinese Spring; DW durum wheat cv. Svevo; WEW, wild emmer wheat.

We applied a whole‐genome alignment approach to compare the three *Aegilops* species and the hexaploid wheat cv. Chinese Spring (CS). *Aegilops longissima* and *Ae. sharonensis* both showed the best alignment to the wheat D subgenome, whereas the *Ae. speltoides* genome had the best alignment to the wheat B subgenome, and in both cases the alignment was linear (Figure 2). To further address the phylogenetic placement of the three *Aegilops* species, we analyzed their high‐confidence genes together with high‐confidence genes from *Ae. tauschii* (a descendant of the donor of the D subgenome), and the subgenomes of CS (A, B and D) and WEW (A and B). Using orthofinder (Emms and Kelly, 2019) (Table S5), we determined orthologous groups across the gene sets and computed a consensus phylogenomic tree based on all clusters. This analysis showed the evolutionary proximity of the *Ae. speltoides* genome to the wheat B subgenome (Figure 3) and the proximity of *Ae. longissima* and *Ae. sharonensis* to *Ae. tauschii* and the wheat D subgenome. These findings further demonstrate the evolutionary relationship between *Ae. speltoides* and the B genome, establishing it as the closest known relative of the wheat B subgenome.

**Figure 4:**
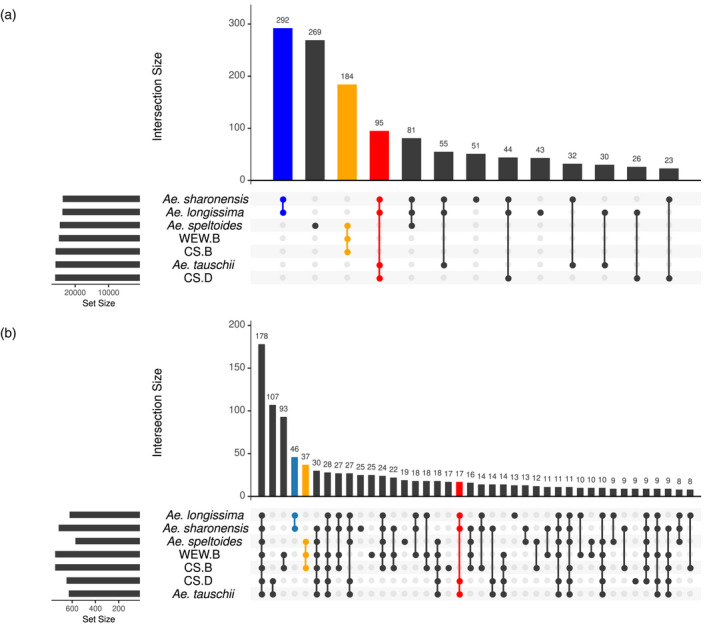
Composition of shared and unique orthogroups between different plant species and subgenomes. The upset plot shows the number of associated orthogroups in each species and shared between species. The upper histogram shows the number of orthogroups (from the orthofinder analysis) for the different combinations. The species/subgenome combination is shown by the dots on the bottom panel. The side histogram shows the total number of orthogroups per species/subgenome. The blue bar highlights the relatively large number of orthogroups shared by *Aegilops longissima* and *Aegilops sharonensis*; the orange bar shows orthogroups shared between *Aegilops speltoides* and the CS and WEW B subgenomes; the red bar shows orthogroups with genes shared between *Ae. longissima*, *Ae. sharonensis*, *Aegilops tauschii* and the CS D subgenome. (a) Orthogroups of all genes for single species or specific species combinations. (b) Orthogroups of *NLR* genes for single species or specific species combinations. CS, Chinese Spring bread wheat; WEW, wild emmer wheat.

The orthologous gene groups obtained by orthofinder were further analysed for clusters with genes shared between the *Aegilops* species and the wheat subgenomes or genes that are unique to a single species or subgenome. Figure 4(a) (‘Upset plot’ of the orthogroups) shows the number and composition of shared and unique orthogroups for the included species and subgenomes. A relatively high number of orthogroups (292) with genes only from both *Ae. longissima* and *Ae. sharonensis* was identified (blue color). Across the B genome group (*Ae. speltoides* and the WEW and CS B subgenomes) there were 184 orthogroups (orange color), and only 95 orthogroups were shared exclusively between the D genome group (*Ae. longissima*, *Ae. sharonensis*, *Ae. tauschii* and the CS D subgenome; red color). These orthologous groups contain potential candidates for group‐specific genes or distinct fast‐evolving genes, and their higher number in the B genome group reflects the known evolutionary distances and time scales in wheat.

Gene alignment between *Ae. longissima* and *Ae. sharonensis* showed a 99% median sequence similarity, and the alignment of either species to the wheat subgenomes showed a median value of 97.3% for the D subgenome genes and 96.8% for the B subgenome genes. The alignment of *Ae. speltoides* high‐confidence genes with the CS and WEW B genome genes had a median value of 97.3%, similar to that of *Ae. longissima* and *Ae. sharonensis* and the wheat D subgenome genes (Figure S3). The complete gene annotation provided here and its relationship to wheat will be a useful resource to locate candidate genes and to target specific genes or gene families for research purposes and for breeding.

### Analysis of haplotype blocks between *Aegilops* and wheat species

**Figure 5:**
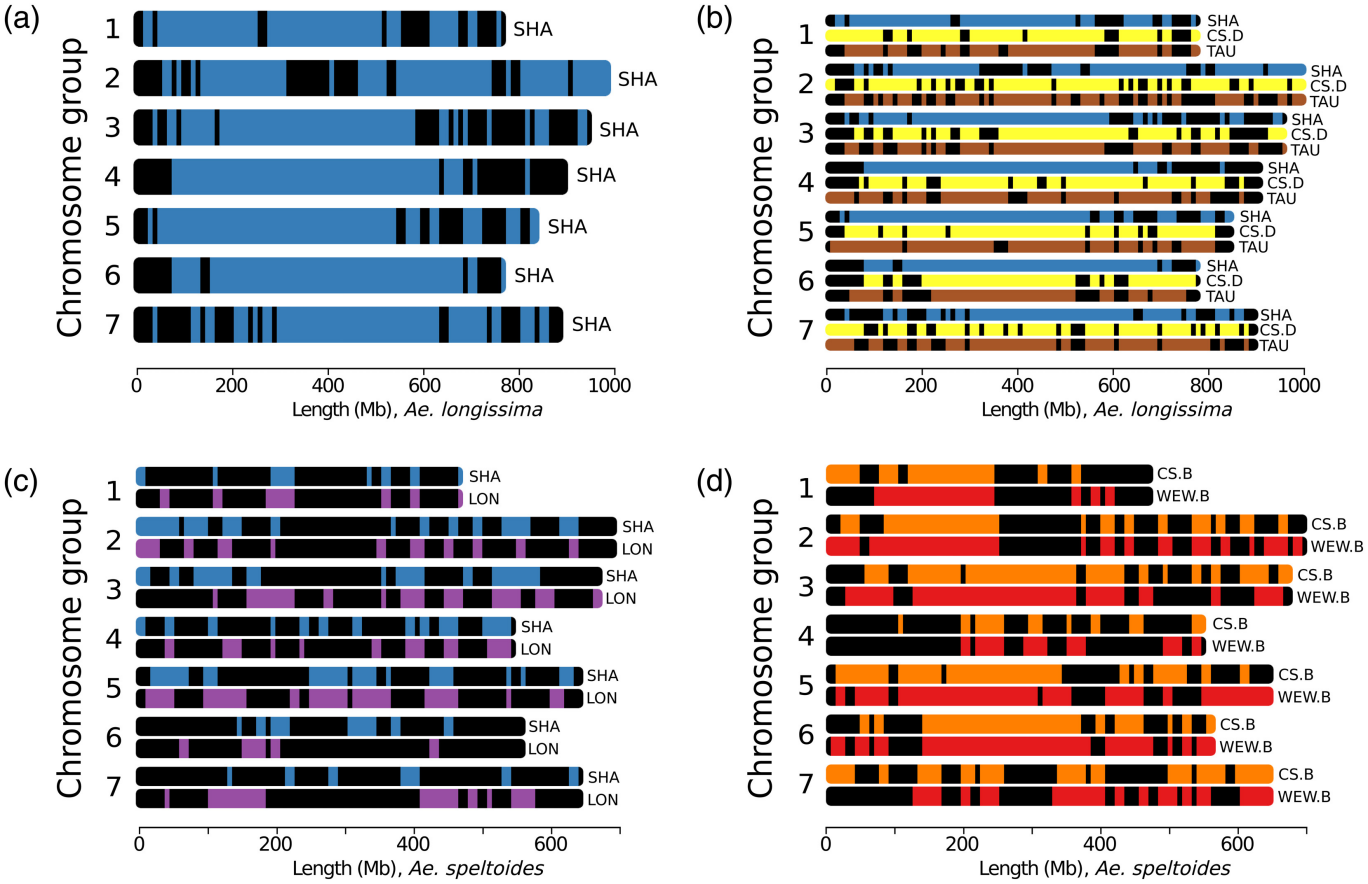
Haploblocks between *Aegilops* and wheat species. Haploblocks represent long shared genome sequences between two or more species at a defined percentage identity. (a) Identified haploblocks between *Aegilops sharonensis* and *Aegilops longissima*. Blue blocks show regions of *Ae. sharonensis* compared with *Ae. longissima* with a median identity of >99% along a 5‐Mb region. (b) Identified haploblocks between *Ae. sharonensis*, CS D, *Aegilops tauschii* and *Ae. longissima*. Blocks show regions with a median identity of >95% (>99% for *Ae. sharonensis* versus *Ae. longissima*) along a 5‐Mb region. Top (blue), *Ae. sharonensis*; middle (yellow), CS D; bottom (brown), *Ae. tauschii*. (c) Identified haploblocks between *Ae. sharonensis*, *Ae. longissima* and *Ae. speltoides*. Blocks show regions with a median identity of >95% along a 5‐Mb region. Top (blue), *Ae. sharonensis*; bottom (purple), *Ae. longissima*. (d) Identified haploblocks between WEW B, CS B and *Aegilops speltoides*. Blocks show regions with a median identity of >95% along a 5‐Mb region. Top (orange), CS; bottom (red), WEW. CS, Chinese Spring bread wheat; LON, *Ae. longissima*; SHA, *Ae. sharonensis*; TAU, *Ae. tauschi*; WEW, wild emmer wheat. The reference genome is indicated by the *x*‐axis label.

We used the strategy described for wheat genomes (Brinton et al., 2020) to construct and define haplotype blocks (haploblocks) within the *Aegilops* species as well as between wheat relatives. In our approach, we used nucmer (Delcher et al., 2002) to compute pairwise alignments between whole chromosomes of the respective genomes and discarded alignments smaller than 20 000 bp. We calculated the percentage identity for each alignment and binned them by chromosomal position in 5‐Mb bins and then combined adjacent bins sharing identical median percentage identity to form a continuous haploblock. We identified haploblocks between the three *Aegilops* species (Figure 1b–d) as well as between four cross‐species sets/combinations: (i) *Ae. sharonensis* and *Ae. longissima* (D genome relatives); (ii) *Ae. longissima*, *Ae. sharonensis*, *Ae. tauschii* and *T. aestivum* D (D genome lineage); (iii) *Ae. sharonensis*, *Ae. longissima* (D genome relatives) and *Ae. speltoides* (B genome relative); and (iv) *Ae. speltoides*, *T. aestivum* B and *T. dicoccoides* B (B genome lineage) (Figure 5). The number of identified haploblocks over all combinations varied between 53 and 93 and cover the entire length of the contiguous sequence, including genes and transposable elements. A summary of all haploblocks and their statistics is provided in Data S1.

We identified 67 haploblocks with an average length of 64 Mb and a similarity of 99% between the highly similar *Ae. longissima* and *Ae. sharonensis* genomes (Figure 5a). The longest haploblocks were identified on chromosomes 4S and 6S. Furthermore, 93 haploblocks ranging between 70 Mb on chromosome 7S and 250 Mb on chromosome 3S were identified between *T. aestivum* D and *Ae. longissima* with a similarity of 95%. Using a similarity cut‐off value of 95%, 90 haploblocks between *Ae. tauschii* and *Ae. longissima* (Figure 5b) were identified. These ranged between 85 Mb on chromosome 1S and 210 Mb on chromosome 3S.

For *Ae. speltoides* and the two other *Aegilops* species, we used a similarity cut‐off value of 95% and identified 58 blocks with *Ae. sharonensis* and 53 blocks with *Ae. longissima* with an overall good positional correlation (Figure 5c) and average lengths of 14 and 17 Mb, respectively.

For the B genome relatives, we identified 63 haploblocks between *T. aestivum* B and *Ae. speltoides* and 56 haploblocks between *T. dicoccoides* B and *Ae. speltoides* (Figure 5d) using a similarity cut‐off value of 95%. Average haploblocks lengths were 29 and 39 Mb, respectively.

In general, these blocks showed a higher positional correlation when compared with the blocks identified between the three newly sequenced *Aegilops* species. These findings indicate larger relative distances between the three *Aegilops* species under investigation with respect to conserved haploblocks, compared with the distances observed within the wheat B and D genome lineages.

This observation is also supported by the higher overall percentage of haploblocks covering the genome: around 20% among *Ae. speltoides*, *Ae. longissima* and *Ae. sharonensis*, but more than double among the D genome relatives and among the B genome relatives.

The defined haploblocks will enable the identification of diverse and similar genetic regions between the different *Aegilops* species and wheat, and will assist breeding efforts by allowing more targeted selection and providing convenient access to previously unused sources of genetic diversity.

### NLR repertoire in *Aegilops* spp.

The Sitopsis species, in particular *Ae. Longissima, Ae. sharonensis* and *Ae. speltoides*, show pronounced variation in resistance against major diseases of wheat (Anikster et al., 2005; Olivera et al., 2007). As the majority of cloned disease‐resistance genes encode NLRs (Kourelis and Van Der Hoom, 2018), we decided to catalog all *NLR*s present in the different genomes, analyse their genomic distribution (Figure 1f), and study their phylogenetic relationships in *Aegilops* and wheat. To identify the *NLR* complement in the different genomes, we: (i) searched the existing annotations of our genome assemblies for disease‐resistance gene analogs; and (ii) performed a *de novo* annotation using nlr‐annotator (Steuernagel et al., 2020) (Table S6). For our final list of *NLR*s, we compared the results of the two types of analyses and selected only the *NLR*s that were found by both methods.

**Figure 6:**
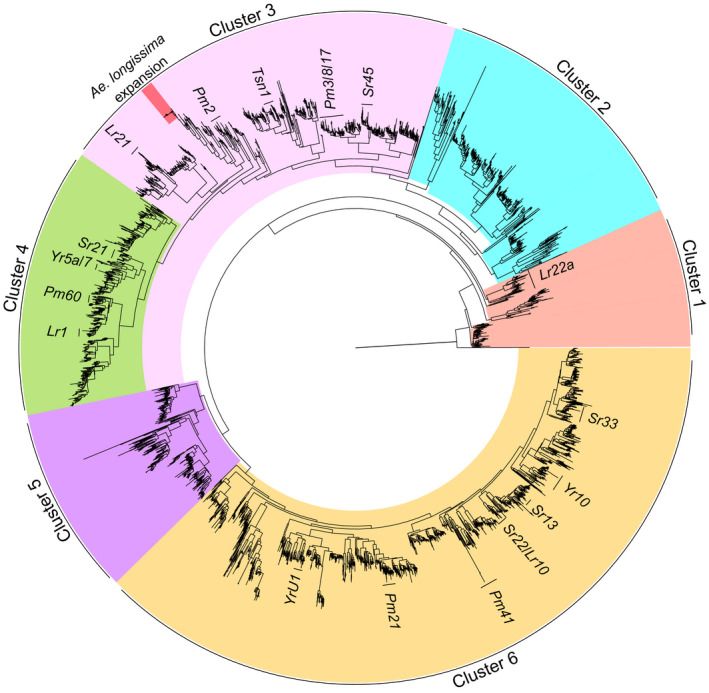
Phylogenetic tree of *NLR* genes in *Aegilops* and wheat. The tree illustrates NLR diversity and the similarity of the genes to cloned genes from wheat. The six clusters represent the major supergroups using an independent clustering analysis. Cluster 3 has a unique *NLR* expansion in *Aegilops longissima* ‘chrUn’ between *Pm2* and *Lr21* containing 32 predicted genes, and this unique *NLR* expansion is homologous to the CS gene ‘*TraesCS2B02G046000.1*’ on chromosome 2B. Another expansion on *Aegilops sharonensis* ‘chrUn’ that contains 11 predicted genes is located on the same branch as the *Tsn1* gene. CS, Chinese Spring bread wheat (see also Figure S4).

The list of candidate *NLR*s that were predicted by both methods contained 742, 800, 1030 and 2674 candidate *NLR*s in the *Ae. longissima*, *Ae. sharonensis*, *Ae. speltoides* and CS genomes, respectively (Table S6). To show the diversity of the *NLR* repertoire in the three *Aegilops* species, we constructed a phylogenetic tree using all of the predicted *NLR*s along with 20 cloned *NLR*‐type resistance genes (Figure 6; Table S7). As expected, in most cases the nearest *NLR* gene to a cloned gene was from CS, but in the case of *YrU1*, *Sr22*, *Lr22a*, *Pm2* and *Pm3*, we also found homologs from the three *Aegilops* species (Figure S4). Two large clades were completely devoid of cloned genes (Figure 6, clusters 2 and 5). These clades might contain *NLR*s that are associated with resistance to pathogens or pests to which no resistance genes have yet been cloned in wheat and its wild relatives.

We used orthofinder software to identify orthologous *NLR*s (ortho*NLR*s) between the six genomes and found 1312 ortho*NLR* groups, of which 112 were species specific. In addition, we found 42 single‐copy ortho*NLR*s (378 predicted genes) that have one copy of each *NLR* present once in each of the nine diploid genomes (we split WEW into A and B subgenomes and CS into A, B and D subgenomes). These single‐copy ortho*NLR*s are attractive targets for the assessment of their association with resistance to specific diseases and for downstream breeding applications. An overview of the distribution of the different ortho*NLR* groups identified in this analysis is presented in Figure 4(b). Notably, 46 *NLR* groups were specific to *Ae. sharonensis* and *Ae. longissima* (Figure 4b, blue bar), highlighting a potential reservoir of species‐specific resistance genes. An additional 37 groups are specific to the *Ae. speltoides*/EmmerB/WheatB B lineage (Figure 3b, yellow bar) and 17 NLR clusters are unique to the *Ae. sharonensis*/*Ae. longissima*/*Ae. tauschii*/WheatD D lineage (Figure 3b, red bar), both of which are likely to represent B and D lineage‐specific *NLR* genes and gene variants.

A phylogenetic tree derived from the ortho*NLR*s (Figure S5; excluding the A subgenome of CS and WEW) is congruent with the species tree obtained from orthologous group clustering of all genes (Figure 4), and highlights the relationships of bread wheat subgenomes and the genomes of the wheat wild relatives. Based on the ortho*NLR* analysis, we identified all the predicted single‐copy *NLR*s between *Ae. sharonensis*, *Ae. longissima* and *Ae. speltoides*. We found 129 single‐copy ortho*NLR*s between the three species, 57 of which were associated with specific haploblocks, with a tendency to cluster towards distal chromosomal regions (Figure 5).

## DISCUSSION

Species in the genus *Aegilops* are closely related to wheat and have high genetic diversity that can potentially be used in wheat improvement. High‐quality reference genome sequences are essential for the efficient exploitation of these genetic resources and can also help to elucidate the evolutionary and genomic relationships of these species and wheat. To this end, we sequenced and assembled the *Ae. longissima* and *Ae. speltoides* genomes and analysed them together with the recently sequenced *Ae. sharonensis* genome (Yu et al., 2021).

Construction of genome assemblies using the tritex pipeline (Monat et al., 2019) resulted in high‐quality pseudomolecule assemblies, as confirmed by busco (96–98% complete genes) and whole‐genome alignment with CS. Notably, the assembled genome size of *Ae. longissima* (6.70 Gb) and *Ae. sharonensis* (6.71 Gb) is substantially larger than that of *Ae. speltoides* (5.14 Gb); the genome of *Ae. speltoides* is similar in size to the published wheat subgenomes (Avni et al., 2017; IWGSC et al., 2018; Maccaferri et al., 2019) and a bit larger than the *Ae. tauschii* (4.0 Gb) genome. The relative total length of the pseudomolecule assemblies of the *Ae. speltoides* genome is similar to measurements of nuclear DNA amount (Eilam et al., 2008). Despite the relatively large genome sizes of *Ae. longissima* and *Ae. sharonensis*, their gene numbers are similar to the number of genes in all other diploid Triticeae genomes.

Species within the genus *Aegilops* have been considered the main donors of wheat diversity. *Aegilops tauschii* is the direct progenitor of the wheat D subgenome, and *Aegilops* species within the section Sitopsis (S genome) were considered potential ancestors of the B genome (Kerby and Kuspira,, 1987). Later studies suggested that *Ae. speltoides* is associated with the B genome, although it is not the direct progenitor of the wheat B subgenome (Badaeva et al., 1996a, 1996b; Maestra and Naranjo, 1998). In contrast to earlier assessments, genomic data associated the remaining Sitopsis species with the wheat D rather than the wheat B subgenome (Marcussen et al., 2014; IWGSC, 2014; Petersen et al., 2006; Yamane and Kawahara, 2005). Our analysis of the three new *Aegilops* reference genomes supports this evolutionary model and provides conclusive and quantitative evidence for the closer association of *Ae. speltoides* to the wheat B subgenome and of *Ae. longissima* and *Ae. sharonensis* to *Ae. tauschii* and the wheat D subgenome.

The best whole‐genome sequence alignment of *Ae. speltoides* with the CS B subgenome (Figure 2) and the relatively high number of shared orthogroups between *Ae. speltoides* and the CS and WEW B subgenomes (184 shared orthogroups) place *Ae. speltoides* in a ‘B’ lineage, together with the WEW and hexaploid wheat B subgenomes, whereas *Ae. longissima* and *Ae. sharonensis* (292 shared orthogroups) are placed in a separate ‘D’ lineage, together with *Ae. tauschii* and the wheat D subgenome (95 shared orthogroups). Accordingly, *Ae. speltoides* should be placed in a phylogenetic group outside the Sitopsis, possibly together with *Amblyopyrum muticum* (syn. *Aegilops mutica*; diploid T genome) (Bernhardt et al., 2020; Edet et al., 2018; Glémin et al., 2019; Huynh et al., 2019). The genome sequences also reveal the extremely high degree of similarity between *Ae. longissima* and *Ae. sharonensis*: the two species have an almost identical genome size and they share 292 orthogroups, compared with only 51 and 43 *Ae. longissima‐* and *Ae. sharonensis*‐specific groups, respectively. In fact, the only substantial genomic rearrangement between the two genomes is the unique 4S–7S translocation in *Ae. longissima*. Despite their highly similar genomes the two species usually occupy different habitats, but are occasionally found in mixed populations that can result in hybrids (Ankori and Zohary, 1962). The accessions of *Ae. longissima* and *Ae. sharonensis* used in this study were both collected in the same region, and genotyping‐by‐sequencing (GBS) analysis showed high similarity between accessions from this area. Accessions from regions with less species overlap show more genetic differentiation (Sela et al., 2018). The two species demonstrate high variability in traits, such as resistance/tolerance to biotic and abiotic stresses (Millet, 2007). The high genome similarities between *Ae. longissima* and *Ae. sharonensis* along with the high phenotypic variability can be used to facilitate the identification of unique traits found in these species.

The new reference genome sequences of the three *Aegilops* species are expected to advance the study and utilization of these species for wheat improvement. To make these fully accessible as resources for targeted breeding, it is necessary to unlock their genetic diversity. To this end, we constructed whole‐genome haploblocks, which facilitate the localization of useful variation on a genome‐wide scale (Brinton et al., 2020). Large consistent haploblocks at 99% identity were observed between the *Ae. longissima* and *Ae. sharonensis* genomes, further confirming the high similarity of these genomes, whereas the number of haploblocks between the three *Aegilops* species is much smaller. These findings once again demonstrate the high association between *Ae. longissima* and *Ae. sharonensis*, and further support the differentiation of *Ae. speltoides* from the Sitopsis clade. Importantly, the haploblocks reveal diverse and non‐diverse regions between the genomes, which points to orthologous genes with potentially beneficial variation that can be accessed by means of wide crossing or transformation.

The three *Aegilops* species contain many attractive traits, and it is expected that the new genome sequences will facilitate the cloning of desired genes. For example, the *Ae. sharonensis* genome encodes resistance against a wide range of diseases that attack wheat (Khazan et al., 2020; Olivera et al., 2007; Millet et al., 2014, 2017; Yu et al., 2017). To better evaluate the genetic diversity and potential of disease‐resistance genes in the three species, we cataloged and analyzed their *NLR* complements, the major class of genes encoded by plant disease‐resistance genes. Additionally, *NLR* genes are largely host specific and are considered the fastest evolving genes (van de Weyer et al., 2019), therefore this group of genes can shed light on recent evolutionary events. A high proportion of the *NLR* genes mapped to the telomere regions, which are also the most differentiated regions between the genomes. However, mapping the high‐confidence genes between *Ae. longissima* and *Ae. sharonensis* showed a mean identity value of 98.6%, whereas the mapping of *NLR* genes showed only 87% identity, suggesting that the combined diversity of disease‐resistance genes present in both genomes is substantially greater than that in each of the single genomes.

Phylogenetic analysis of the NLRs from the different genomes outlined the diversity in this class of proteins and highlighted groups of genes or specific targets for further study, for example, in the two branches of the *NLR* phylogeny that lacked cloned resistance genes (clusters 2 and 5; Figure 6). Alternatively, clades rich in cloned genes (such as clusters 3, 4 and 6) can be considered evolutionary hotspots. The phylogenetic tree and the orthogroup analysis both provided a cross reference to locate orthologous *NLR*s in CS and the three *Aegilops* species, such as the CS *Pm2* gene, which is located on a branch that contains *NLR*s from all of the three subgenomes as well as from the three *Aegilops* species (Figure S4). Another example is the gene expansion in *Ae. longissima* that corresponds to the CS gene ‘*TraesCS2B02G046000.1*’ on chromosome 2B at position 23 020 418–23 022 244 bp on the CS genome. This locus also coincides with the *MlIW39* powdery mildew resistance locus on the short arm of chromosome 2B (Qiu et al., 2021). This cluster of *NLR*s is located on chrUn in *Ae. longissima*, so its exact genomic location remains to be defined. This expansion spreads over several scaffolds; therefore, it is probably not a sequencing artifact.

Recent advances in sequencing and genomic‐based approaches have greatly enhanced the identification and cloning of new genes, thus expediting the sourcing of candidate genes for next‐generation breeding (cloned gene table within Gaurav et al., 2021; Hafeez et al., 2021). The genome sequences of the three *Aegilops* species reported here reveal important details of the genetic make‐up of these species and their association with durum and bread wheat. These new discoveries and the availability of high‐quality reference genomes pave the way for a more efficient utilization of these species, which have long been recognized as important genetic resources for wheat improvement.

## EXPERIMENTAL PROCEDURES

### Plant materials

*Aegilops longissima* accession AEG‐6782‐2 was collected from Ashdod, Israel (31.84°N, 34.70°E). *Aegilops speltoides* ssp. *speltoides* accession AEG‐9674‐1 was collected from Tivon, Israel (32.70°N, 35.10°E). Each accession was self‐pollinated for four generations to increase homozygosity. All accessions were propagated and maintained at the Lieberman Okinow gene bank at the Institute for Cereal Crops Improvement at Tel Aviv University.

Leaf samples were collected from seedlings grown at 22 ± 2°C for 2–3 weeks with a 12‐h light/12‐h dark regime in fertile soil. Before harvesting leaves, the plants were maintained for 48 h in a dark room to lower the levels of plant metabolites. Samples were collected directly before extraction.

### Isolation of high‐molecular‐weight DNA

High‐molecular‐weight DNA was extracted using the liquid nitrogen grinding protocol (BioNano Genomics, https://bionanogenomics.com/wp‐content/uploads/2018/02/30177‐Bionano‐Prep‐Plant‐Tissue‐DNA‐Isolation‐Liquid‐Nitrogen‐Grinding‐Protocol.pdf) following the protocol described by Zhang et al. (1995), with modifications as follows. All steps were performed in a fume hood on ice using ice‐cold solutions. Approximately 1 g of fresh leaf tissue was placed in a Petri dish with 4 ml of nuclear isolation buffer (NIB, 10 m m Tris–HCl, pH 8, 10 m m EDTA, 80 m m KCl, 0.5  m sucrose, 1 m m spermidine, 1 m m spermine, 8% PVP) and cut into 2 × 2‐mm pieces using a razor blade. The volume of NIB was brought to 10 ml, and the material was homogenized using a Tissue Ruptor (cat. no. 9001271; QIAGEN, https://americanlaboratorytrading.com/lab‐equipment‐products/qiagen‐tissueruptor_10681) for 60 sec. Then, 3.75 ml of β‐mercaptoethanol and 2.5 ml of 10% Triton X‐100 were added, and the homogenate was filtered through a 100‐μm filter (cat. no. 21008‐950; VWR, https://www.vwr.com), followed by washing three times with 1 ml of NIBM (NIB supplemented with 0.075% β‐mercaptoethanol). The homogenate was filtered through a 40‐μm filter (cat. no. 21008‐949; VWR), the volume of the filtrate was adjusted to 45 ml by adding NIBTM (NIB supplemented with 0.075% β‐mercaptoethanol and 0.2% Triton‐X 100) and the mixture was centrifuged at 2000 ***g*** for 20 min at 4°C. The nuclear pellet was resuspended in 1 ml of NIBM, and NIBTM was added to a final volume of 4 ml. The nuclei were layered onto cushions made of 5 ml of 70% Percoll (cat. no. P1644; Merck, https://www.sigmaaldrich.com) in NIBTM and centrifuged at 600 ***g*** for 25 min at 4°C in a swinging bucket centrifuge. The pelleted nuclei were washed once by resuspension in 10 ml of NIBM, centrifuged at 2000 ***g*** for 25 min at 4°C, washed three times each with 10 ml of NIBM and then centrifuged at 3000 ***g*** for 25 min at 4°C. The pelleted nuclei were resuspended in 200 μl of NIB and mixed with a 140‐μl aliquot of melted 2% low‐melting‐point agarose (cat. no. 1703594; Bio‐Rad, https://www.bio‐rad.com) at 43°C, and the mixture was solidified in 50‐μl plugs on an ice‐cold casting surface. DNA was released by digestion with ESSP (0.1  m EDTA, 1% sodium lauryl sarcosine, 0.2% sodium deoxycholate, 1.48 mg ml^−1^ proteinase K) for 36 h at 50°C followed by RNase (cat. no. 158924; QIAGEN, https://www.qiagen.com) treatment and extensive washes. High‐molecular‐weight DNA was stored in Tris‐EDTA buffer at 4°C without degradation for up to 8 months.

### Sequencing

The 470‐bp (250 paired‐end) *Ae. longissima* libraries were sequenced by Novogene (https://en.novogene.com) on an Illumina HiSeq 2500 (https://www.illumina.com). The libraries were sequenced at the Roy J. Carver Biotechnology Center at University of Chicago (UC), Illinois. For both *Ae. longissima* and *Ae. speltoides*, 9‐kb mate‐pair libraries (150 paired‐end) were generated and sequenced at the Roy J. Carver Biotechnology Center at UC Illinois. The 10x Genomics chromium libraries (https://www.10xgenomics.com) were prepared for each genotype following the Chromium Genome library protocol v2 (10x Genomics) and sequenced at the Genome Canada Research and Innovation Centre using the manufacturer’s recommendations across two lanes of Illumina HiSeqX with 150‐bp paired‐end reads to a minimum 30× coverage. FASTQ files were generated by longranger (10x Genomics) for analysis (Walkowiak et al., 2020). Hi‐C libraries were prepared at the Genome Center at the Leibniz Institute for Plant Genetics and Crop Plant Research, Gatersleben, Germany, using previously described methods (Beier et al., 2017). Raw data and pseudomolecule sequences were submitted to the European Nucleotide Archive (https://www.ebi.ac.uk/ena), as described in Table S8.

### Assembly

The tritex pipeline (Monat et al., 2019) was used for genome assembly. Raw data and pseudomolecule sequences were deposited at the European Nucleotide Archive (Table S8). As a result of the high residual heterozygosity in chromosomes 1S and 4S of *Ae. speltoides*, *de novo* assembly of a Hi‐C map did not yield satisfactory results for these two chromosomes, which had to be ordered and oriented solely by collinearity to the bread wheat cv. Chinese Spring B genome (IWGSC et al., 2018).

### Annotation

Structural gene annotation was performed according to the method previously described by Monat et al. (2019) using *de novo* annotation and homology‐based approaches with RNA‐seq data sets generated for *Ae. sharonensis* (Yu et al., 2021). Annotation files for the three *Aegilops* genomes are available to download (https://doi.org/10.5447/ipk/2022/0).

Using evidence derived from expression data, RNA‐seq data were first mapped using hisat 2.0.4 (Kim et al., 2015) (parameter: ‐‐dta) and subsequently assembled into transcripts by stringtie 1.2.3 (Pertea et al., 2015) (parameters: ‐m 150‐t ‐f 0.3). *Triticeae* protein sequences from available public data sets (UniProt, 5 October 2016) were aligned against the genome sequence using genomethreader 1.7.1 (Gremme et al., 2005) (arguments: ‐startcodon ‐finalstopcodon ‐species rice ‐gcmincoverage 70 ‐prseedlength 7 ‐prhdist 4). All transcripts from RNA‐seq and aligned protein sequences were combined using cuffcompare 2.2.1 (Ghosh and Chan, 2016) and subsequently merged with stringtie 1.2.3 (parameters: ‐‐merge ‐m150) into a pool of candidate transcripts. transdecoder 3.0.0 was used to find potential open reading frames and to predict protein sequences within the candidate transcript set.

*Ab initio* annotation using augustus 3.3.2 (Stanke et al., 2006) was performed to further improve structural gene annotation. To avoid potential over‐prediction, we generated guiding hints using the above RNAseq, protein evidence and transposable element predictions. A specific model for *Aegilops* was trained using the steps provided in Hoff and Stanke (2019) and later used for prediction. All structural gene annotations were joined using evidencemodeller (Haas et al., 2008), and weights were adjusted according to the input source: *ab initio* (2); homology based (5). Additionally, two rounds of program to assemble spliced alignments (pasa) (Haas et al., 2003) were run to identify untranslated regions and isoforms using transcripts generated by a genome‐guided trinity (Grabherr et al., 2011) assembly derived from *Ae. sharonensis* RNA‐seq data.

We used BLASTP (Altschul et al., 1990) (ncbi‐blast‐2.3.0+, parameters ‐max_target_seqs 1 ‐evalue 1e–05) to compare potential protein sequences with a trusted set of reference proteins (Uniprot Magnoliophyta, reviewed/Swissprot, downloaded on 3 August 2016). This differentiated candidates into complete and valid genes, non‐coding transcripts, pseudogenes and transposable elements. In addition, we used PTREP (release 19; http://botserv2.uzh.ch/kelldata/trep‐db/index.html), a database of hypothetical proteins containing deduced amino acid sequences in which internal frameshifts have been removed in many cases. This step is particularly useful for the identification of divergent transposable elements with no significant similarity at the DNA level. Best hits were selected for each predicted protein to each of the three databases. Only hits with an E‐value below 10e–10 were considered. Furthermore, only hits with subject coverage (for protein references) or query coverage (transposon database) above 95% were considered significant, and protein sequences were further classified using the following confidence: a high‐confidence protein sequence was complete and had a subject and query coverage above the threshold in the UniMag database, or no BLAST hit in UniMag but a hit in UniPoa (https://www.uniprot.org) and not PTREP; a low‐confidence protein sequence was incomplete and had a hit in the UniMag or UniPoa databases, but not in TREP. Alternatively, it had no hit in UniMag, UniPoa or PTREP, but the protein sequence was complete. The tag REP was assigned for protein sequences not in UniMag, but was complete and had hits in PTREP.

Functional annotation of predicted protein sequences was carried out using the ahrd pipeline (https://github.com/groupschoof/AHRD). busco (Simão et al., 2015) was used to evaluate the gene space completeness of the pseudomolecule assembly with the ‘embryophyta_odb10’ database containing 1614 single‐copy genes.

### Genome alignments

The *Aegilops* genomes were aligned with CS using minimap 2 (Li, 2018) (https://github.com/lh3/minimap2). The aligned genome was split into 1‐kb blocks using the bedtools (Quinlan and Hall, 2010) makewindows command (https://bedtools.readthedocs.io/en/latest/) and then aligned with the CS genome. Results were filtered to include only alignments with a Mapping Quality of 60 and a minimal length of 750 bp.

### NLR annotations

nlr‐annotator (Steuernagel et al., 2020) was used to locate all *NLR* sequences in the three genomes. An NB‐ARC domain global alignment was created through the pipeline from a subset of complete *NLR*s. Known *NLR* resistance genes were used as a reference for the tree (Table S8). fasttree 2 (Price et al., 2010) (http://www.microbesonline.org/fasttree) was used to generate a phylogenetic tree from all the NB‐ARC sequences. As the nlr‐annotator pipeline uses NP_001021202.1 (Cell Death Protein 4, CDP4) as an outgroup, we used NP_001021202.1 for tree rooting. We also extracted genes from the whole‐genome shotgun sequence annotation by the assigned function of ‘Disease’, ‘NBS‐LRR’ or ‘NB‐ARC’, and these were matched with the nlr‐annotator annotation by position using the intersect option in bedtools (Quinlan and Hall, 2010).

Protein sequences of all NLRs were also clustered using cd‐hit (Limin et al., 2012) (https://github.com/weizhongli/cdhit). All the large clusters (*n* > 30) were located on the tree, and only six large clusters were chosen to represent major branches.

### Phylogeny/orthologs

orthofinder (Emms and Kelly, 2019) (https://github.com/davidemms/OrthoFinder) was used to locate orthologous genes between the *Aegilops* and wheat annotations, and only high‐confidence genes were used. We used the three *Aegilops* annotations, *Ae. tauschii*, the AB annotation of WEW and the ABD annotation of CS. The polyploid annotations (CS and WEW) were split into the subgenomes, and each was handled separately (Table S9). Additionally, orthofinder was used to identify orthologous *NLR* sequences (that were a subset from the gene annotation) and to analyze the phylogenetic relationship between the genomes and subgenomes. To compare the gene annotations, BLASTN was used to align the high‐confidence genes from *Ae. longissima* and *Ae. sharonensis* with each other and with the B and D subgenomes of CS and high‐confidence genes from *Ae. speltoides* to the B subgenomes of CS and WEW.

### Haploblocks

Haploblock analysis was performed using the methods described by Brinton et al. (2020). Only alignments larger than 10 kb were kept and spurious alignments were discarded. Further modifications were made in terms of a lowered percentage identity for some pairwise comparisons to reflect the phylogenetic distance.

## AUTHOR CONTRIBUTIONS

Extracted DNA for whole‐genome shotgun sequencing and long mate‐pair libraries: AM‐D, JD and HS. 10x Chromium sequencing: JE and CP. Hi‐C sequencing: AH and NS. Contribution of the *Ae. sharonensis* genome: GY and BBHW. Prepared material and involved in *Ae. speltoides* sequencing: HS, AD and CP. Assembled the *Ae. longissima* and *A. speltoides* genomes: MM and RA. Gene annotation: TL, HG and MS. Performed bioinformatics analyses including whole‐genome alignment, haploblocks, orthofinder and busco: TL, MS and RA. Performed NLR analysis: RA, TL, BS and HS. Provided scientific support: EM and KFXM. Conceived study: AS, RA, BBHW and HS. Drafted manuscript: RA, AS and BBHW. All co‐authors read and approved the final article for publication. Authors are grouped by institution in the author list, except for the first four and the last four authors.

## CONFLICT OF INTEREST

The authors declare that they have no conflicts of interest associated with this work.

## Supporting information

**Figure S1**. Hi‐C contact matrices for *Aegilops longissima* and *Aegilops speltoides*.
**Figure S2**. busco assessment of the completeness of the gene annotation using the ‘embryophyta_odb10’ database, which includes 1614 core plant genes (*32*).
**Figure S3**. Gene annotation pair alignments.
**Figure S4**. Detailed phylogenetic tree of *NLR* genes in *Aegilops* and wheat.
**Figure S5**. Phylogenetic tree generated from the orthofinder analysis with all nlr‐annotator gene predictions as input.
**Table S1**. Overview of the three *Aegilops* assemblies showing all chromosomes, including chromosome ‘Un’ with all unassociated scaffolds.
**Table S2**. Contig assembly details.
**Table S3**. GC percentage per chromosome compared with the subgenomes of *Triticum aestivum*.
**Table S4**. Transposon composition (percentage of the genome) compared with the subgenomes of *Triticum aestivum*.
**Table S5**. Summary of orthofinder results for all high‐confidence genes.
**Table S6**. Number of predicted *NLR* genes in different genomes.
**Table S7**. Cloned *NLR* genes* used as reference for the *NLR* phylogenetic tree.
**Table S8** Project numbers for European Nucleotide Archive raw sequencing data and pseudomolecule submission.
**Table S9**. Number of high‐confidence genes per species used for ortholog phylogenetic analysis.

**Data S1**. Summary of haploblocks with reference *Aegilops tauschii*.

## Data Availability

The data sets generated during and/or analyzed during the current study are publicly available. The sequence reads and the genome assemblies were deposited in the European Nucleotide Archive under project numbers PRJEB41661, PRJEB41746, PRJEB40543, PRJEB40544, PRJEB40050 and PRJEB40051. Gene annotation files are available to download (http://dx.doi.org/10.5447/ipk/2022/0). Seeds of *Ae. longissima* accession AEG‐6782‐2 and *Ae. speltoides* accession AEG‐9674‐1 are available from the Institute for Cereal Crops Improvement at Tel Aviv University (https://en‐lifesci.tau.ac.il/icci).
