# Supplementary material for: Genome sequences of three Aegilops species of the section Sitopsis reveal phylogenetic relationships and provide resources for wheat improvement
Source: Plant J. 2022 Feb 12;110(1):179–92. doi: 10.1111/tpj.15664 (PMC10138734; doi:10.1111/tpj.15664)
Supplement: Supplementary file 2 — Figure S1. Hi‐C contact matrices for Aegilops longissima and Aegilops speltoides. Figure S2. busco assessment of the completeness of the gene annotation using the ‘embryophyta_odb10’ database, which includes 1614 core plant genes (32). Figure S3. Gene annotation pair alignments. Figure S4. Detailed phylogenetic tree of NLR genes in Aegilops and wheat. Figure S5. Phylogenetic tree generated from the orthofinder analysis with all nlr‐annotator gene predictions as input. Table S1. Overview of the three Aegilops assemblies showing all chromosomes, including chromosome ‘Un’ with all unassociated scaffolds. Table S2. Contig assembly details. Table S3. GC percentage per chromosome compared with the subgenomes of Triticum aestivum. Table S4. Transposon composition (percentage of the genome) compared with the subgenomes of Triticum aestivum. Table S5. Summary of orthofinder results for all high‐confidence genes. Table S6. Number of predicted NLR genes in different genomes. Table S7. Cloned NLR genes* used as reference for the NLR phylogenetic tree. Table S8 Project numbers for European Nucleotide Archive raw sequencing data and pseudomolecule submission. Table S9. Number of high‐confidence genes per species used for ortholog phylogenetic analysis. [file TPJ-110-179-s001.docx]

**Supporting information**

Supporting information file includes:

Figs. S1 to S5

Tables S1 to S9

Other Supplementary Materials for this manuscript include the following:

Table S4 (as a detailed high resolution PDF)

Supplementary dataset 1


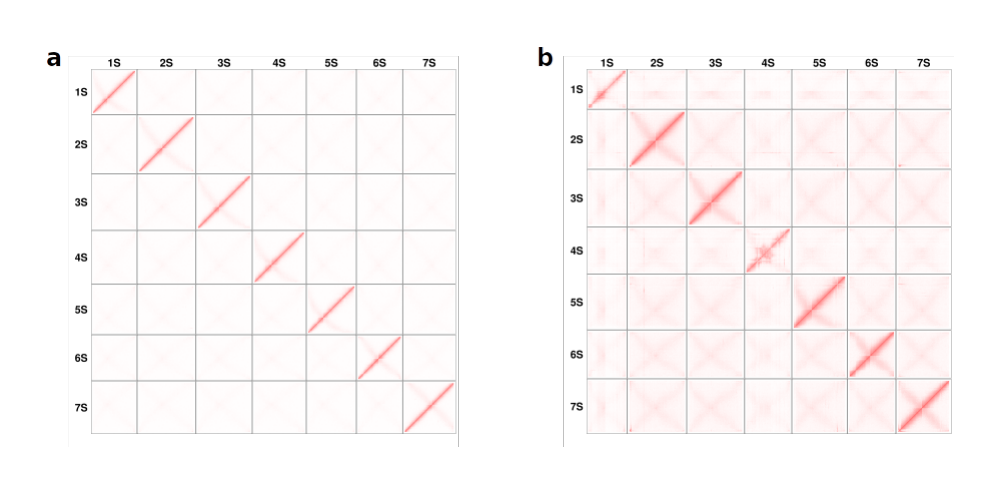


Figure S1. Hi-C contact matrices for a, *Ae. longissima* and b, *Ae. speltoides*.


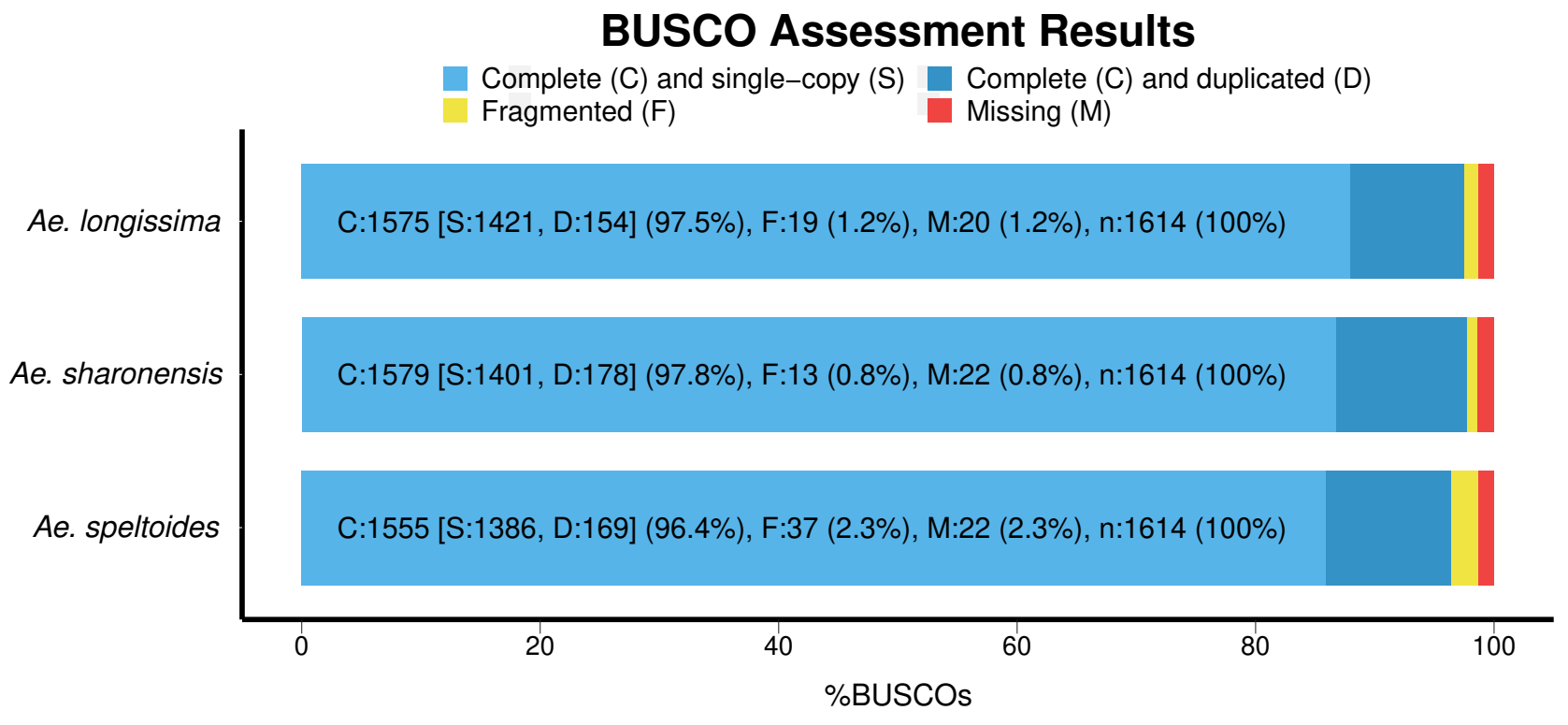


Figure S2. BUSCO assessment of the completeness of the gene annotation using the ‘embryophyta_odb10‘ database that includes 1,614 core plant genes (*32*).


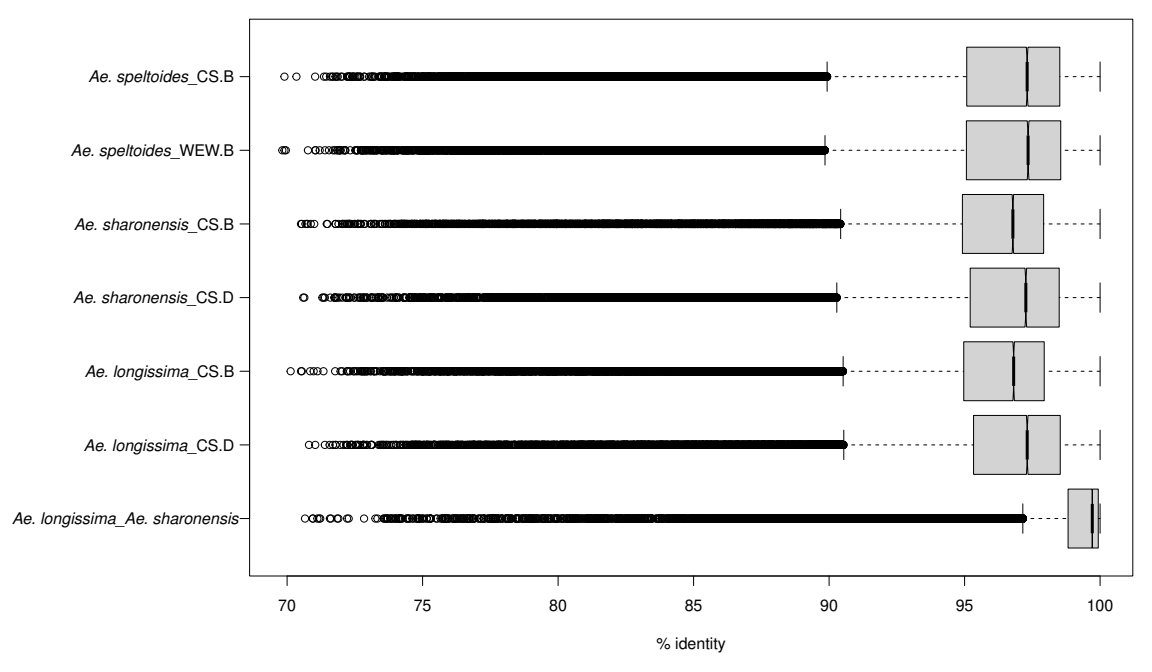


Figure S3. Gene annotation pair alignments. Box plots show the percentage of identity between the best hit for each query (first label name) gene in the reference (second label name) annotation.

Figure S4. This figure is included as a separate PDF file. Detailed phylogenetic tree of NLR genes in *Aegilops* and wheat. Cloned genes (Table S7) were added to the tree, and their branches are highlighted. Tip labels refer to NLR-Annotator IDs. Tip point color shows whether a match is found between the NLR annotation (NLR-Annotator) and the whole-genome annotation; gray point means no match. WEW, wild emmer wheat, *Triticum turgidum* ssp. *dicoccoides*. CS, bread wheat, *Triticum aestivum* cv. Chinese Spring.


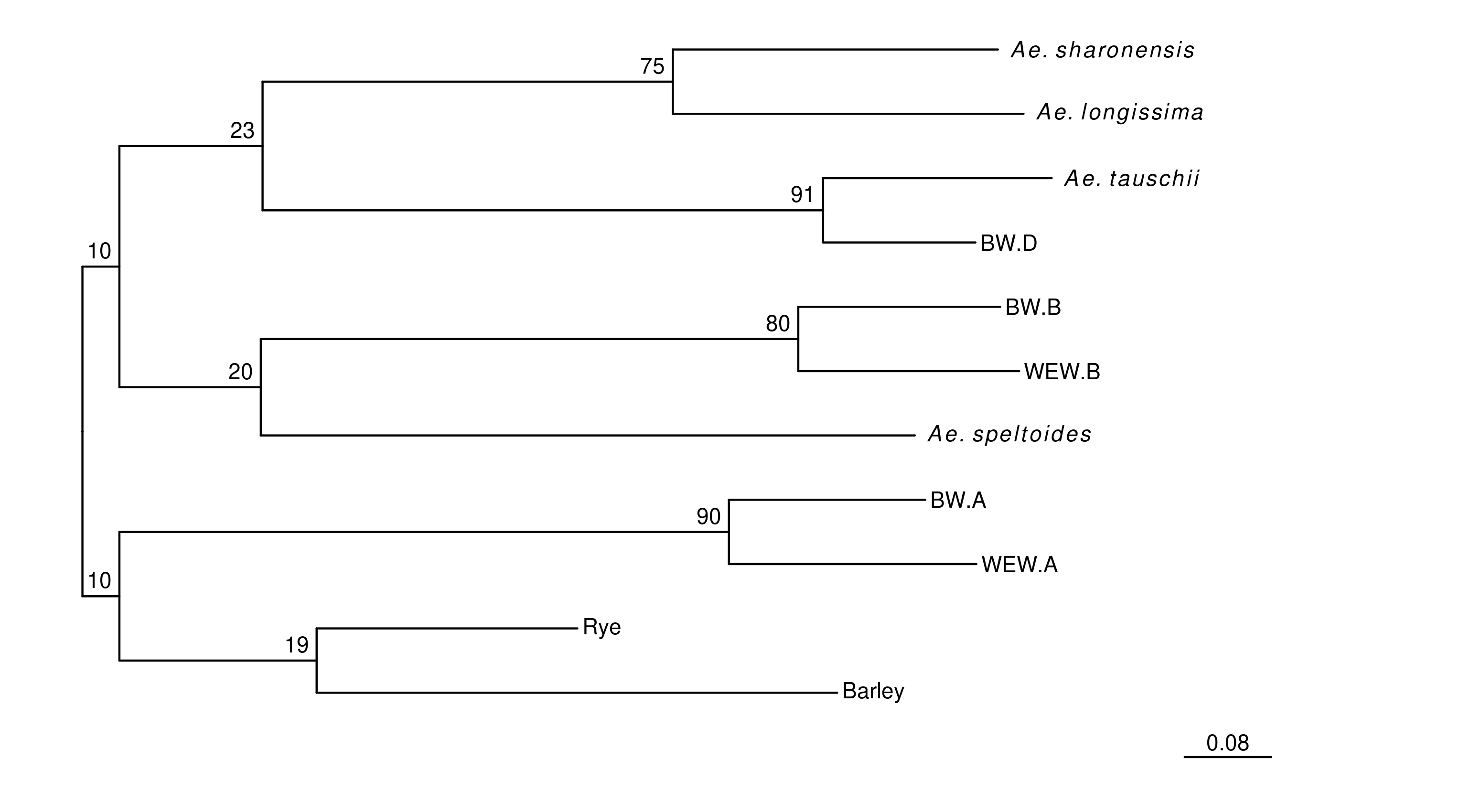


**Figure S5.** Phylogenetic tree generated from the OrthoFinder analysis with all NLR-Annotator gene predictions as input. Values correspond to OrthoFinder-based support values.

Table S1 Overview of the three *Aegilops* assemblies showing all chromosomes including chromosome “Un” with all unassociated scaffolds.

| *Ae. longissima* |  |  |  |  |  |  |  |
| --- | --- | --- | --- | --- | --- | --- | --- |
| Chromosome | Chromosome length (bp) | ^a^No. HC genes | ^b^No. LC genes | Total No. genes | N50 (bp) | N90 (bp) | #scaffolds |
| 1S | 742,303,966 | 3,438 | 3,973 | 7,411 | 4,561,840 | 1,138,021 | 289 |
| 2S | 956,173,857 | 4,644 | 5,107 | 9,751 | 4,849,770 | 1,135,822 | 362 |
| 3S | 916,702,776 | 4,531 | 4,957 | 9,488 | 4,807,476 | 999,978 | 368 |
| 4S | 874,517,040 | 3,697 | 4,426 | 8,123 | 4,768,090 | 1,074,529 | 346 |
| 5S | 816,294,110 | 4,328 | 4,595 | 8,923 | 4,330,990 | 1,052,266 | 347 |
| 6S | 750,216,944 | 3,259 | 4,082 | 7,341 | 3,936,747 | 1,048,299 | 312 |
| 7S | 862,608,691 | 3,680 | 4,814 | 8,494 | 4,285,042 | 1,113,450 | 339 |
| Un | 783,655,401 | 3,606 | 4,956 | 8,562 | 56,180 | 1,491 | 127,984 |
| Total | 6,702,472,785 | 31,183 | 36,910 | 68,093 | 3,754,329 | 242,603 | 130,347 |
| *Ae. speltoides* |  |  |  |  |  |  |  |
| Chromosome | Chromosome length (bp) | ^a^No. HC genes | ^b^No. LC genes | Total No. genes | N50 (bp) | N90 (bp) | #scaffolds |
| 1S | 445,829,560 | 3,178 | 4,484 | 7,662 | 1,338,029 | 378,510 | 529 |
| 2S | 657,893,865 | 5,522 | 6,750 | 12,272 | 7,145,516 | 1,121,567 | 281 |
| 3S | 636,117,214 | 5,137 | 6,359 | 11,496 | 14,542,279 | 1,329,581 | 209 |
| 4S | 520,569,408 | 3,149 | 4,127 | 7,276 | 2,294,611 | 639,716 | 386 |
| 5S | 614,738,994 | 5,113 | 6,471 | 11,584 | 9,396,929 | 1,445,106 | 203 |
| 6S | 536,175,046 | 3,861 | 5,350 | 9,211 | 6,487,485 | 1,624,515 | 188 |
| 7S | 610,578,938 | 4,538 | 6,477 | 11,015 | 7,894,947 | 1,022,973 | 264 |
| Un | 1,114,993,717 | 6,430 | 18,109 | 24,539 | 21,791 | 1,622 | 185,157 |
| Total | 5,136,896,742 | 36,928 | 58,127 | 95,055 | 3,111,390 | 20,000 | 187,217 |
| *Ae. sharonensis*^c^ |  |  |  |  |  |  |  |
| Chromosome^c^ | Chromosome length (bp)^c^ | ^a^No. HC genes | ^b^No. LC genes | Total No. genes | N50 (bp)^c^ | N90 (bp)^c^ | #scaffolds^c^ |
| 1S | 782,818,162 | 3,678 | 4,396 | 8,074 | 14,301,836 | 1,946,926 | 168 |
| 2S | 1,022,071,454 | 5,093 | 5,760 | 10,853 | 12,458,785 | 2,598,021 | 182 |
| 3S | 971,920,087 | 4,983 | 5,579 | 10,562 | 17,681,665 | 2,964,762 | 161 |
| 4S | 827,198,496 | 3,298 | 4,016 | 7,314 | 17,868,812 | 2,716,220 | 138 |
| 5S | 867,619,200 | 4,713 | 4,961 | 9,674 | 13,883,202 | 3,670,029 | 126 |
| 6S | 806,566,123 | 3,599 | 4,568 | 8,167 | 13,434,032 | 2,801,261 | 123 |
| 7S | 1,015,700,474 | 4,710 | 6,119 | 10,829 | 9,208,414 | 1,394,866 | 268 |
| Un | 420,139,911 | 1,124 | 2,200 | 3,324 | 15,399 | 1,188 | 93,480 |
| Total | 6,714,033,907 | 31,198 | 37,599 | 68,797 | 12,378,845 | 1,132,218 | 94,646 |

^a^HC, high-confidence genes.

^b^LC, low-confidence genes.

^C^Adapted from^29^.

**Table S2** Contig assembly details.

|  | *Ae. longissima* | *Ae. speltoides* |
| --- | --- | --- |
| Number of contigs | 2,889,061 | 630,998 |
| Assembly size (bp) | 6,669,327,844 | 4,242,364,366 |
| Largest contig (bp) | 130,822 | 204,857 |
| N50 (bp) | 8,705 | 15,594 |
| N90 (bp) | 640 | 2,501 |
| Number of reads PE450 | 1,799,826,940 | 1,657,407,912 |
| Number of reads MP9 | 2,546,743,998 | 5,972,478,314 |
| Number of reads 10X | 1,728,375,244 | 1,607,543,608 |
| Number of reads HiC | 580,092,794 | 407,339,022 |
| Total number of reads | 6,655,038,976 | 9,644,768,856 |
| Size of reads PE450 (bp) | 449,956,735,000 | 435,798,323,050 |
| Size of reads MP9 (bp) | 380,174,931,236 | 890,913,303,977 |
| Size of reads 10X (bp) | 260,984,661,844 | 242,739,084,808 |
| Size of reads HiC (bp) | 58,589,372,194 | 41,141,241,222 |
| Total size of reads (bp) | 1,149,705,700,274 | 1,610,591,953,057 |
| Genome coverage (1x) | 171.535 | 313.534 |

**Table S3** GC percent per chromosome compared to the subgenomes of *T. aestivum.*

| **Chr** | *Ae. longissima* | *Ae. sharonensis* | *Ae. speltoides* | *Ae.* *tauschii* | *T. aestivum* D genome | *T. aestivum* B genome | *T. aestivum* A genome |
| --- | --- | --- | --- | --- | --- | --- | --- |
| **1** | 46.8 | 46.8 | 47.3 | 46.3 | 46.3 | 46.1 | 45.8 |
| **2** | 46.8 | 46.8 | 46.9 | 46.4 | 46.3 | 46.1 | 45.9 |
| **3** | 46.8 | 46.7 | 46.9 | 46.4 | 46.3 | 46.2 | 45.8 |
| **4** | 47.0 | 47.1 | 47.3 | 46.6 | 46.5 | 46.3 | 45.9 |
| **5** | 46.8 | 46.7 | 47.0 | 46.4 | 46.2 | 46.1 | 45.8 |
| **6** | 46.9 | 46.8 | 47.0 | 46.5 | 46.4 | 46.2 | 45.9 |
| **7** | 46.8 | 46.7 | 46.9 | 46.3 | 46.2 | 46.1 | 45.7 |
| **Un** | 46.3 | 46.3 | 47.6 | 44.6 |  |  |  |

**Table S4** Transposon composition (percentage of the genome) compared to the subgenomes of *T. aestivum*.

|  | *Ae. longissima* | *Ae. sharonensis* | *Ae. speltoides* | *Ae.* *tauschii* | *T. aestivum* D genome | *T. aestivum* B genome | *T. aestivum* A genome |
| --- | --- | --- | --- | --- | --- | --- | --- |
| Mobile Element (TXX) | 82.5 | 82.3 | 78.7 | 80.5 | 80.4 | 82.3 | 82.6 |
| Class I: Retroelement (RXX) | 70.9 | 70.7 | 69.9 | 64.2 | 64.8 | 69.3 | 71.8 |
| LTR Retrotransposon (RLX) | 70.5 | 70.4 | 69.4 | 63.8 | 64.4 | 68.9 | 71.5 |
| Ty1/copia (RLC) | 15.5 | 15.5 | 18.3 | 15.8 | 15.7 | 16.7 | 16.3 |
| Ty3/gypsy (RLG) | 33.8 | 33.7 | 30.8 | 26.9 | 27.2 | 32.4 | 32.5 |
| unclassified LTR (RLX) | 21.2 | 21.2 | 20.3 | 21.1 | 21.5 | 19.8 | 22.7 |
| non-LTR Retrotransposon (RXX) | 0.4 | 0.4 | 0.5 | 0.4 | 0.4 | 0.4 | 0.3 |
| LINE (RIX) | 0.3 | 0.3 | 0.5 | 0.4 | 0.4 | 0.4 | 0.3 |
| SINE (RSX) | 0.0 | 0.0 | 0.0 | 0.0 | 0.0 | 0.0 | 0.0 |
| Class II: DNA Transposon (DXX) | 11.1 | 11.1 | 8.3 | 15.9 | 15.2 | 12.5 | 10.3 |
| DNA Transposon Superfamily (DTX) | 11.0 | 11.0 | 8.1 | 15.7 | 15.0 | 12.4 | 10.2 |
| CACTA superfamily (DTC) | 10.7 | 10.6 | 7.7 | 15.3 | 14.6 | 12.0 | 9.8 |
| hAT superfamily (DTA) | 0.01 | 0.01 | 0.01 | 0.00 | 0.00 | 0.01 | 0.00 |
| Mutator superfamily (DTM) | 0.15 | 0.15 | 0.14 | 0.20 | 0.20 | 0.15 | 0.14 |
| Tc1/Mariner superfamily (DTT) | 0.03 | 0.03 | 0.04 | 0.04 | 0.04 | 0.03 | 0.04 |
| PIF/Harbinger (DTH) | 0.10 | 0.10 | 0.13 | 0.11 | 0.12 | 0.11 | 0.10 |
| unclassified (DTX) | 0.05 | 0.05 | 0.06 | 0.06 | 0.06 | 0.05 | 0.06 |
| DNA Transposon Derivative (DXX) | 0.11 | 0.11 | 0.14 | 0.13 | 0.13 | 0.11 | 0.11 |
| MITE (DXX) | 0.11 | 0.11 | 0.14 | 0.13 | 0.13 | 0.11 | 0.11 |
| Helitron (DHH) | 0.01 | 0.01 | 0.01 | 0.02 | 0.01 | 0.01 | 0.01 |
| unclassified DNA transposon (DXX) | 0.01 | 0.01 | 0.01 | 0.01 | 0.01 | 0.01 | 0.01 |
| Unclassified Element (TXX) | 0.48 | 0.48 | 0.55 | 0.45 | 0.45 | 0.44 | 0.40 |

*Transposons have been annotated by homology to a Triticeae TE library as described in (Maccaferri *et al.,* 2019).

**Table S5** Summary of OrthoFinder results for all high-confidence genes.

|  | Number of genes | Number of genes in orthogroups | Number of unassigned genes | % of genes in orthogroups | % of unassigned genes | Number of orthogroups containing species | % of orthogroups containing species | Number of species-specific orthogroups | Number of genes in species-specific orthogroups | % of genes in species-specific orthogroups |
| --- | --- | --- | --- | --- | --- | --- | --- | --- | --- | --- |
| *Ae. longissima* | 31,183 | 30,902 | 281 | 99.1 | 0.9 | 23,633 | 72 | 15 | 102 | 0.3 |
| *Ae. sharonensis* | 31,198 | 30,961 | 237 | 99.2 | 0.8 | 23,559 | 71.8 | 14 | 40 | 0.1 |
| *Ae. speltoides* | 36,928 | 36,327 | 601 | 98.4 | 1.6 | 24,427 | 74.4 | 136 | 1,083 | 2.9 |
| WEW.A | 32,706 | 31,943 | 763 | 97.7 | 2.3 | 25,222 | 76.9 | 69 | 158 | 0.5 |
| WEW.B | 33,052 | 32,222 | 830 | 97.5 | 2.5 | 24,679 | 75.2 | 129 | 353 | 1.1 |
| CS.A | 35,275 | 34,656 | 619 | 98.2 | 1.8 | 26,354 | 80.3 | 58 | 132 | 0.4 |
| CS.B | 35,561 | 34,903 | 658 | 98.1 | 1.9 | 25,706 | 78.3 | 46 | 101 | 0.3 |
| CS.D | 34,161 | 33,627 | 534 | 98.4 | 1.6 | 25,813 | 78.7 | 34 | 79 | 0.2 |
| *Ae. tauschii* | 39,622 | 37,318 | 2304 | 94.2 | 5.8 | 25,720 | 78.4 | 510 | 2,398 | 6.1 |

WEW, wild emmer wheat, *Triticum turgidum* ssp. *dicoccoides*.

CS, bread wheat, *Triticum aestivum* cv. Chinese Spring.

**Table S6** Number of predicted NLR genes in different genomes.

| Species | ^a^NLR-Annotator | ^b^PGSB annotation |
| --- | --- | --- |
| *Ae. sharonensis* | 1,451 (979) | 1,143 (773) |
| *Ae. longissima* | 1,555 (978) | 1,065 (732) |
| *Ae. speltoides* | 1,358 (721) | 1,570 (1,045) |
| *Ae. tauschii* | 888 |  |
| *T. aestivum* (CS) | 3,184 (A, 1,064; B, 1,198; D, 922) |  |
| *T. dicoccoides* (WEW) | 2,230 (A, 1,015; B, 1,215) |  |

^a^Number represents all predicted genes. The number in parentheses represents only complete genes.

^b^Number represents disease resistance + NBS-NLR + NB-ARC genes. The number in parentheses represents only NBS-NLR + NB-ARC genes.

WEW, wild emmer wheat, *Triticum turgidum* ssp. *dicoccoides*.

CS, bread wheat, *Triticum aestivum* cv. Chinese Spring.

**Table S7** Cloned NLR genes* used as reference for the NLR phylogenetic tree.

| **Gene** | **Associated Phenotype** | **Gene class** | **Genome** | **Origin** | **Reference** | **DOIs** |
| --- | --- | --- | --- | --- | --- | --- |
| *Lr1* | Leaf rust resistance | NLR | 5D | *Ae. tauschii* | Cloutier et al. 2007 | doi.org/10.1007/s11103-007-9201-8 |
| *Lr10* | Leaf rust resistance | NLR | 1A | Wheat | Feuillet et al. 2002 | doi.org/10.1046/j.1365-313X.1997.11010045.x |
| *Lr21* | Leaf rust resistance | NLR | 1D | *Ae. tauschii* | Huang et al. 2003 | PMC1462593 |
| *Lr22a* | Leaf rust resistance | NLR | 2D | Bread wheat | Thind et al. 2017 | doi.org/10.1038/nbt.3877 |
| *Pm2* | Powdery mildew resistance | NLR | 5D | Bread wheat | Sánchez-Martín et al. 2016 | doi.org/10.1186/s13059-016-1082-1 |
| *Pm3* | Powdery mildew resistance | NLR | 1A | Wheat | Yahiaoui et al. 2004 | doi.org/10.1046/j.1365-313X.2003.01977.x |
| *Pm8* | Powdery mildew resistance | NLR | 1B/1R | Wheat/Rye | Hurni et al. 2013 | doi.org/10.1111/tpj.12345 |
| *Pm17* | Powdery mildew resistance | NLR | 1B/1R | Wheat/Rye | Singh et al. 2018 | doi.org/10.1007/s11103-018-0780-3 |
| *Pm21* | Powdery mildew resistance | NLR | 6V | *Haynaldia villosa* | Xing et al. 2017 | doi.org/10.1016/j.molp.2018.02.013 |
| *Pm41* | Powdery mildew resistance | NLR | 3B | WEW | Li et al. 2020 | doi.org/10.1111/nph.16761 |
| *Pm60* | Powdery mildew resistance | NLR | 7A | *T. urartu* | Zou et al. 2017 | doi.org/10.1111/nph.14964 |
| *Sr13* | Stem rust resistance | NLR | 6A | Durum wheat | Zhang et al. 2017 | doi.org/10.1073/pnas.1706277114 |
| *Sr21* | Stem rust resistance | NLR | 2A | *T. monococcum* | Chen et al. 2018 | doi.org/10.1371/journal.pgen.1007287 |
| *Sr22* | Stem rust resistance | NLR | 7A | *Triticum boeoticum* | Steuernagel et al. 2016 | doi.org/10.1038/nbt.3543 |
| *Sr33* | Stem rust resistance | NLR | 1D | *Ae. tauschii* | Periyannan et al. 2013 | doi.org/10.1126/science.1239028 |
| *Sr45* | Stem rust resistance | NLR | 1D | *Ae. tauschii* | Steuernagel et al. 2016 | doi.org/10.1038/nbt.3543 |
| *Tsn1* | Septoria nodorum blotch and tan spot | NLR | 5B | Wheat | Faris et al. 2010 | doi.org/10.1073/pnas.1004090107 |
| *Yr5a* (*Yr5*) | Stripe rust resistance | BED-NLR | 2B | Bread wheat | Marchal et al. 2018 | doi.org/10.1038/s41477-018-0236-4 |
| *Yr7* | Stripe rust resistance | BED-NLR | 2B | Bread wheat | Marchal et al. 2018 | doi.org/10.1038/s41477-018-0236-4 |
| *YrU1* | Stripe rust resistance | NLR | 5A | *T. urartu* | Wang et al. 2020 | doi.org/10.1038/s41467-020-15139-6 |

*Adapted from (Gaurav *et al.* 2021).

WEW, wild emmer wheat, *Triticum turgidum* ssp. *dicoccoides*.

**Table S8** Project numbers for European Nucleotide Archive raw sequencing data and pseudomolecule submission.

| Species | Type | Project ID |
| --- | --- | --- |
| *Aegilops longissima* accession AEG-6782-2 | All raw data (PE, MP, 10X) | PRJEB41661 |
| *Aegilops speltoides* var. *speltoides* accession AEG-9674-1 | All raw data (PE, MP) | PRJEB41746 |
| *Aegilops speltoides* var. *speltoides* accession AEG-9674-1 | 10X | PRJEB48314 |
| *Aegilops longissima* accession AEG-6782-2 | Hi-C | PRJEB40543 |
| *Aegilops speltoides* var. *speltoides* accession AEG-9674-1 | Hi-C | PRJEB40544 |
| *Aegilops longissima* accession AEG-6782-2 | Pseudomolecules | PRJEB40050 |
| *Aegilops speltoides* var. *speltoides* accession AEG-9674-1 | Pseudomolecules | PRJEB40051 |

Table S9 Number of high-confidence genes per species used for ortholog phylogenetic analysis.

| *Ae. tauschii* | 39,622 |
| --- | --- |
| *Ae. longissima* | 31,183 |
| *Ae. sharonensis* | 31,198 |
| *Ae. speltoides* | 36,928 |
| WEW_A | 32,706 |
| WEW_B | 33,052 |
| CS_A | 35,275 |
| CS_B | 35,561 |
| CS_D | 34,161 |

WEW, wild emmer wheat, *Triticum turgidum* ssp. *dicoccoides*.

CS, bread wheat, *Triticum aestivum* cv. Chinese Spring.
