# Supplementary material for: Genome sequences of three Aegilops species of the section Sitopsis reveal phylogenetic relationships and provide resources for wheat improvement
Source: Plant J. 2022 Feb 12;110(1):179–92. doi: 10.1111/tpj.15664 (PMC10138734; doi:10.1111/tpj.15664)
Supplement: Supplementary file 1 [file TPJ-110-179-s003.pdf]

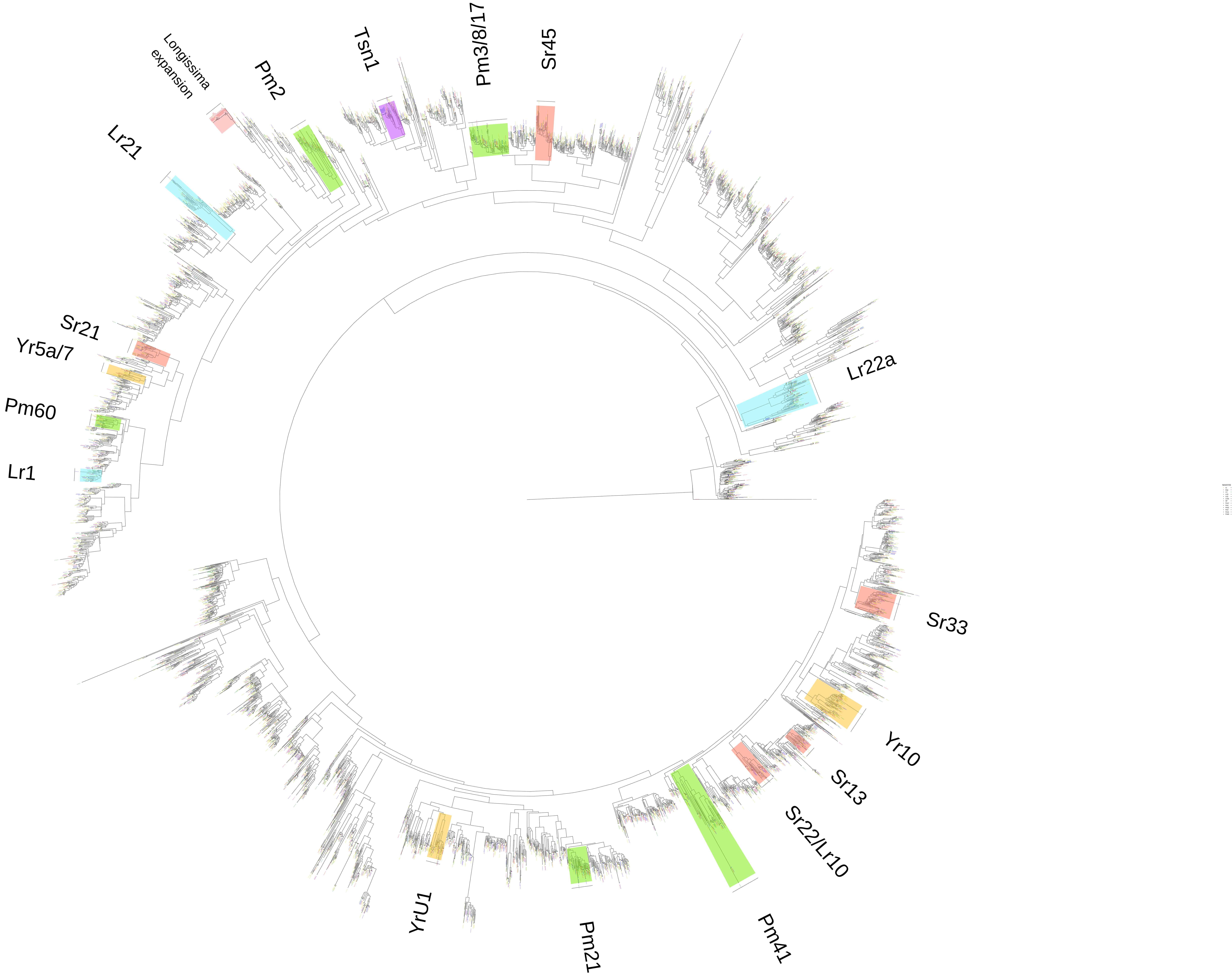
Longissima expansion
Pm2
Tsn1
Pm3/8/17
Sr45
Lr21
Sr21
Yr5a/7
Pm60
Lr1
Lr22a
Sr33
Yr10
Sr13
Sr22/Lr10
Pm41
Pm21
YrU1
